# Casemix-based economic incentives that work

**DOI:** 10.1186/1472-6963-11-S1-A19

**Published:** 2011-10-19

**Authors:** M Hansen, PE Hansen

**Affiliations:** 1 Ministry of Interior and Health, Denmark, Copenhagen, 1216, Denmark

## Introduction

Since 2001, the Danish healthcare system has been characterized by a focus on the reduction of waiting lists. By introducing activity-based funding of the regions and hospitals, there has been an increase in activity, a reasonably positive development in productivity, a high degree of patient and citizen satisfaction, and a successful reduction of waiting lists. One of the reasons for this success is that it was possible to use Casemix-based economic incentives actively.

## Methods

Over the last few years, this development has been supplemented by a wish to prevent and avoid unnecessary admission to hospitals by including the municipalities as active participants in the healthcare sector. This has been done by using a regulation that makes the municipalities co-financing partners of the regions and hospitals. Since 2012, they have had to pay almost 20% of the regional budget as activity-based financing. Every time a citizen uses the hospital, or a GP, etc., the municipality has to pay.

Over the same period, there has been discussion about whether it is possible to find incentives that will lead to the proper treatment of a patient. This can be seen as a response to quality problems that persist in clinical practice.

To support this development, there will be some experimentation with Pay-for-Performance (P4P) schemes that tie a portion of provider payments to performance based on measures of quality. Several key issues will be considered in determining the optimal design and implementation methods for P4P programs. These include:

1. Choice of clinical practice area

2. Size of financial incentives and who should receive them

3. Selection of quality measures and performance thresholds that determine incentive eligibility

4. Data collection methods

5. Best mix of financial and non-financial incentives.

## Results

In order to move it onto a politically acceptable path, the financing model in the healthcare sector will be changed accordingly. Economic incentives will be used whenever possible. The introduction of these incentives will be done as part of an evolution of the healthcare system, not as a revolution of the system.

## Conclusions

The Ministry of Interior and Health finds that the use of economic incentives can support the movement of the healthcare sector in a politically specified direction.

